# Abcès para-pharyngé survenant chez un diabétique

**DOI:** 10.11604/pamj.2015.20.114.6130

**Published:** 2015-02-09

**Authors:** Madiha Mahfoudhi, Khamassi Khaled

**Affiliations:** 1 Service de Médecine Interne A, Hôpital Charles Nicolle, Tunis, Tunisie; 2 Service ORL, Hôpital Charles Nicolle, Tunis, Tunisie

**Keywords:** abcès, pharynx, diabète, abscess, pharynx, diabetes

## Image en medicine

Les abcès para-pharyngés surviennent surtout chez les immunodeprimés notamment les diabétiques. Ce sont des infections préstyliennes dont l'origine est le plus souvent dentaire. La TDM est systématique et urgente vu l'intérêt diagnostique, topographique et thérapeutique. Patient âgé de 52 ans diabétique de type II a consulté pour une odynophagie et un torticolis évoluant depuis 3 jours. L'examen physique a objectivé une fièvre à 39°C, des caries dentaires, une tuméfaction cervicale gauche de 4 cm de grand axe, douloureuse avec des signes inflammatoires en regard et des adénopathies centimétriques cervicales et sous maxillaires. L'examen oropharyngé a révélé une tuméfaction de la paroi latéro-pharyngée déjetant l'amygdale en dedans. A la biologie, il avait un syndrome inflammatoire. Son diabète était déséquilibré. Les prélèvements bactériologiques étaient négatifs. La TDM cervicale a objectivé une collection para pharyngée gauche. La TDM thoracique n'a pas trouvé de médiastinite. Le traitement s'est basé sur une antibiothérapie intraveineuse de 15 jours faite d'amoxicilline-acide clavulanique, métronidazole et gentamycine associée à un drainage chirurgical. Une équilibration du diabète était réalisée en parallèle. Des soins dentaires ont été indiqués en vue de prévenir d'autres complications infectieuses sur ce terrain diabétique. L’évolution était favorable sur le plan clinique et biologique.

**Figure 1:**
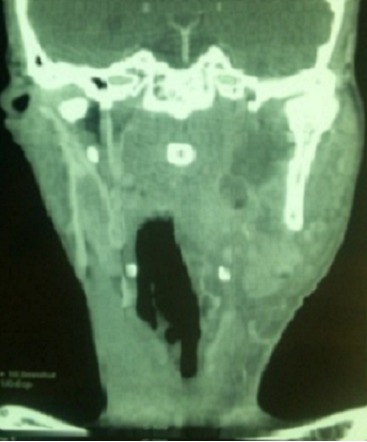
TDM cervicale: collection para-pharyngée gauche

